# Crossroads of Choice: A qualitative study of the factors influencing decisions to transition from sex work among women engaged in sex work in Southern Uganda

**DOI:** 10.21203/rs.3.rs-4468785/v1

**Published:** 2024-06-07

**Authors:** Jennifer Nattabi, Ozge Sensoy Bahar, Josephine Nabayinda, Proscovia Nabunya, Joshua Kiyingi, Samuel Kizito, Flavia Namuwonge, Edward Nsubuga, Susan S. Witte, Fred M. Ssewamala

**Affiliations:** Washington University in St. Louis; Washington University in St. Louis; Washington University in St. Louis; Washington University in St. Louis; Washington University in St. Louis; Washington University in St. Louis; Washington University in St. Louis; Washington University in St. Louis; Columbia University; Washington University in St. Louis

**Keywords:** Women Engaged in Sex Work, Transition from sex work, Commercial sex work, Uganda, Sub-Saharan Africa

## Abstract

**Background:**

Women Engaged in commercial Sex Work (WESW) are exposed to behavioral, biological, and structural factors that exacerbate their risk to HIV infection and other sexually transmitted infections. While commercial sex work may appear voluntary, WESW are more likely to be constrained to selling sex due to limited viable alternatives. To effectively support this vulnerable group of women, it is critical to understand factors that facilitate and impede their decisions to transition from sex work into other careers or jobs. The current study explored women’s decision to transition from sex work into other careers or jobs.

**Methods:**

Semi-structured in-depth interviews were conducted with 53 WESW aged 20–47 enrolled within a larger study-Kyaterekera study, a randomized clinical trial (N = 542) implemented in 19 HIV hotspots in the Southern region of Uganda. Participants were selected based on their intervention attendance (high/medium/low attendance). The interviews were conducted in Luganda the widely spoken language in the study area to explore the factors influencing women’s decisions to from transition from sex work to other jobs or careers. The main interview question used for this study was, *“What are some of the factors that may influence whether you would transition from sex work to other jobs or vocations?”*. All interviews were audio-recorded, transcribed verbatim and translated into English. Thematic analysis in Dedoose software was used to analyze the data.

**Results:**

Participants reported three primary types of decisions, including considering leaving sex work, deciding to leave, and continuing sex work. The emerging themes from the interviews were categorized into individual and structural level facilitators and barriers to leave sex work. Individual level factors included issues of stigma, discrimination, and aging as factors that facilitated women’s decision to leave sex work. At the structural level, factors which include interpersonal stigma and discrimination (from immediate family and community members), physical and sexual violence and income related factors were identified as facilitators and barriers to leaving sex work.

**Conclusion:**

Our study highlights the complex decision-making processes among WESW as they navigate transitions to alternative jobs or careers. By advocating for multifaceted interventions and policies tailored to the diverse challenges faced by WESW, our study contributes to a more informed approach to supporting their transition out of sex work.

## Introduction and Background

The health risks and social stigma associated with commercial sex work remain a global public health concern in developed and developing countries (Wilson, 2019). Due to the nature of their work, women engaged in commercial sex work (WESW) experience behavioral (e.g. inconsistent condom use, multiple and concurrent sexual partners, and alcohol and substance use) (Decker et al., 2020; Strathdee et al., 2015); biological (e.g. high prevalence of sexually transmitted infections) (Cimino, 2012; Mazeingia & Negesse, 2020), and structural-level challenges (e.g. poverty, stigma and discrimination, gender inequality, physical and sexual violence) (Kiyingi et al., 2022) that exacerbate their risk to HIV infection and other sexually transmitted infections (STIs).

Commercial sex work encompasses a range of activities that involve providing sexual services in exchange for money or goods. These activities include street-based sex work, escort services, brothel-based sex work, online or virtual sex work, and participation in adult entertainment industries (e.g., pornography) (Harcourt & Donovan, 2005). Poverty and lack of resources have been highlighted as the major drivers of sex work (McCarthy et al., 2014; Pascoe et al., 2015; Scorgie et al., 2013). However, there are several other factors, such as a history of childhood physical and sexual abuse, neglect, drug addiction, and domestic violence (Nabayinda et al., 2023). Despite the negative outcomes, such as the mental and physical health risks associated with sex work (Farley, 2018; Oram et al., 2012; Phrasisombath, Faxelid, et al., 2012; Sanders, 2004), once women get involved, it becomes rather challenging to exit sex work (Mayhew & Mossman, 2007; Williamson & Folaron, 2003). As WESW continue to trade sex, they may be exposed to other problems like interpersonal violence, economic abuse (Jennings Mayo-Wilson et al., 2023), drug addiction, stigma and discrimination, arrest from law enforcement bodies (Mazeingia & Negesse, 2020), and mental health problems such as depression, stress, and anxiety.

While commercial sex work may appear voluntary as a chosen source of income, WESW are more likely to be constrained to selling sex due to limited viable alternatives (Gould & Fick, 2008). The topic about individuals transitioning out of sex work has gained significant attention within academic circles, with an expanding body of literature dedicated to exploring this complex process (Shareck et al., 2020). For instance, a study in the US found that race (being black) and age (entering sex work as a minor) were significant barriers to leaving sex work (Hankel et al., 2016). Another study in India identified key factors that would help women to exit sex work and these included support from social services agencies, positive relationships, employment opportunities, and better economic conditions (Wilson & Nochajski, 2018). The study highlighted that 70% of the women who participated in the study and had exited sex work emphasized the role of social welfare agencies in facilitating change towards their exit.

Several qualitative studies have also examined the experiences, challenges, and successes of women that are involved in exiting sex work (Cascio, 2019; Hamdan, 2022; Heinz, 2020; Mazeingia & Negesse, 2020; Mihretie et al., 2023; Perri et al., 2022). For instance, a qualitative study with 18 female sex workers in Ethiopia, looked at the intentions, barriers, and opportunities to exit commercial sex work (Mazeingia & Negesse, 2020). Results from this study indicated that women engaged in commercial sex work primarily as a means of survival. Moreover, limited access to viable financial alternatives created significant challenges for these women to exit commercial sex work. Similar results were reported in a study with 13 women engaged in street-based sex work in Ethiopia (Yosef & Bihonegn, 2024). The study found that formal support services, support from family, spirituality, and saving money were factors that led to successful exit from street-based sex work. However, structural problems, drug addiction, and societal attitudes were identified as factors that led to re-entry into sex work.

Another study in Rwanda explored women’s experiences of joining and leaving sex work (Ingabire et al., 2012) among 70 female sex workers. Results showed that motivations to leave sex work included encountering a distressing incident, succumbing to peer pressure, and concerns about dependent children. A study that explored the challenges faced by women while attempting to exit “street-based” sex trade in South Africa highlighted the individual (trauma, physical health, fear of shame), relational factors (lack of social support from family and the community, family obligations), and structural factors (economic necessity, lack of alternative employment) as some of the major barriers to exit sex work (Learmonth et al., 2015).

In Uganda, the focus of this study, sex work continues to be illegal and highly stigmatized (Schulkind et al., 2016) compromising WESW’s ability to have access to resources they may need to successfully navigate the challenges associated with commercial sex work, including exiting sex work. Existing studies among WESW in Uganda primarily focus on HIV and STI prevalence and prevention, risk behaviors among this population, risk factors associated with sex work, and reasons for entry in sex work (Bahar et al., 2023; Kiyingi et al., 2022; Nabayinda et al., 2022, 2023). Yet, to our knowledge, no studies have examined the processes and factors related to sex work exit. Hence, this qualitative study serves to fill the gap in the literature and to inform research and practice on the relevant interventions and programs that can be designed to help vulnerable women successfully transition out of sex work.

## Theoretical Framework

This study is guided by Fuchs Ebaugh’s Role Exit Theory. The theory explains how individuals separate themselves or exit from a particular role or social identity, such as exiting prostitution, conviction, or alcoholism (Fuchs, 1988). The theory argues that exiting a role is a gradual process that involves several stages. These stages include a period of disenchantment with the current role, a search for alternative roles, and a period of readjustment to the new role. The theory also posits that exiting a role can be difficult and stressful, as individuals may face resistance from their peers and other community members. This may result in feelings of loss or identity confusion. The theory also suggests that individuals may experience different levels of support and resources during the role exit process, which can affect their ability to successfully transition to a new role. Månsson & Hedin also make a similar point and emphasize that the ability of an individual to transition out of a role, such as sex work in this instance, and harness their creative and innovative potential is contingent upon the presence of dependable social support systems, resources, and supportive institutions within their surroundings (Månsson & Hedin, 1999).

In this study, we applied Role Exit theory to explore the multi-level factors that influence WESW’s decision and unpack how they weigh these different factors against each other as they consider the option to transition from sex work into other jobs or vocations. Women often face considerable challenges in determining the best course of action and understanding the potential psychological, financial, and public perception consequences that may arise from their choices. Internal and external factors, play a role in influencing the actions of women. These factors include receiving comments from others, a strong desire for freedom, fatigue from the job, as well as enduring feelings of shame and stigma (Baker et al., 2010). Therefore, interventions designed to help women transition from sex work should be mindful and considerate of the convoluted process involved in exiting sex work.

## Methods

### Overall Study design

This qualitative study is a component of a randomized clinical trial funded by the National Institute of Mental Health (R01MH116768). The study was designed to evaluate the effectiveness of a combination intervention that integrates microfinance components into traditional HIV risk reduction (HIVRR) strategies on reducing new cases of STIs among 542 WESW across 19 HIV hotspots in the Greater Masaka Region (Ssewamala et al., 2019). Eligibility criteria for WESW to participate in the study included: (1) being 18 years or older; (2) reporting engagement in unsafe transactional sex (defined as a sex act in exchange for pay) in the past 30 days; and (3) reporting involvement in one or more episodes of unprotected sex in the past 30 days (Ssewamala et al., 2019).

The study employed an embedded experimental mixed methods design (Greene et al., 1989), collecting qualitative data across the study arms at three intervention points in time: pre-intervention completion at 6 month (Time 1), at 12-month (Time 2), and 24-month (Time 3) follow-up (Baharet al., 2023).

### Qualitative Sample Selection

The qualitative sampling was based on participants’ attendance of intervention sessions, i.e., HIV risk reduction (4 sessions) and financial literacy training (6 sessions). For each site (n = 19), one participant from the highest, mid (average), and lowest quartiles of the total number of attended sessions was selected (n = 57). For time 2 qualitative data collection, semi-structured in-depth interviews were completed with 53 participants from 19 sites.

### Qualitative Data Collection

Face-to-face semi-structured in-depth interviews were conducted with 53 WESW at 12-month (Time 2) follow-up. Time 2 interviews focused on participants’ living experiences, the difficulties they faced in their daily living situations, their experiences with the intervention and its specific components. Additionally, participants were asked about their financial decision-making and its impact on different aspects of life such as parenting, becoming pregnant, engaging in protected and unprotected sex, and transitioning to different jobs or vocations, among others. The main interview question used for this study was, *“What are some of the factors that may influence whether you would transition from sex work to other jobs or vocations?”*.

Interviews were conducted in Luganda, the local language widely used in the study region. The semi-structured interview guide was translated from Luganda to English and back translated to Luganda by proficient team members. Interview questions were reviewed and revised by the study team, which included research assistants and co-investigators fluent in both languages to ensure that the questions were culturally and contextually appropriate. Interviews were conducted by male and female research assistants fluent in both Luganda and English and trained extensively by two authors with qualitative research expertise. The qualitative interviews time averaged 140 minutes (range 88 to 268 minutes) and were conducted in a private place with only the research assistant and the participant present to avoid any interruptions. All interviews were audiotaped with consent from the participants, and field notes were taken upon completion.

### Qualitative Data Analysis

All interviews were transcribed and translated verbatim to English before exporting to Dedoose software to facilitate the data analysis process. We used thematic analysis to allow us to use the preexisting themes such as structural and individual-level barriers and facilitators (Boyatzis, 1998; Fereday & Muir-Cochrane, 2006; Guest et al., 2006). The data analysis revealed several subthemes related to the decisions of sex workers to consider leaving sex work, the decision-making process involved in leaving sex work, and the factors influencing their choices to remain in sex work. These decisions were influenced by a combination of individual and structural factors. Interview transcripts were initially read multiple times and independently coded by two authors using sensitizing concepts informed by the existing literature as well as identifying emergent themes (open coding) (Strauss & Corbin, 1998). Broader themes were broken down into smaller, more specific units until no further subcategory was necessary. Initial codes were discussed during team meetings and reorganized when necessary to create a final codebook that was used to code all transcripts. The secondary analysis, conducted by two authors, compared and contrasted themes and categories to identify similarities, differences, and relationships among findings. Peer debriefing and audit trail were used to ensure rigor (Deborah, 2006; Lincoln & Guba, 1985). Specifically, the codes and the findings were presented to the study team who were not involved in the data analysis to discuss the plausibility of themes and related findings (Lincoln &Guba, 1985).

## Results

### Participant demographics

Participants’ age ranged between 20 to 47 years (mean age: 31.79) (Table 1). Fifty-eight percent (58%) of the participants were divorced, half of them (50%) belonged to the Catholic religion, and at least 38% had dropped out before completing primary school. In terms of family size, the number of people in a participant’s household ranged from 1 to 8 people, 0 to 6 children under 18 years. Participants’ income ranged between 10,000 to 700,000 Ugandan shillings ($2.74 to $191.78 at a rate of $3,650). All participants were tested for HIV and other STIs and sixty-four percent of the women tested negative for HIV. Participants had spent at least six years in sex work with a mean age of twenty-five years at the time of starting sex work. Participants also reported to have had an average of 28 customers in the last 30 days. To ensure the privacy and confidentiality of participants, pseudonyms are utilized throughout.

### Factors Influencing Women’s Decisions to Transition from Sex Work

Participants reported three main types of decisions regarding their transitioning from sex work: 1) Considering leaving sex work, 2) Leaving sex work, and 3) Continuing with sex work. Among the three types of decisions made by WESW, a range of factors at the individual and structural levels were identified to have influenced the decisions taken. These factors were found to be relevant to all three types of decisions mentioned. Of the 53 women interviewed, 30 reported that they were considering leaving sex work, 6 stated that they had decided to leave sex work, and 17 reported that they had chosen to remain in sex work.

### 1. Individual level factors that influenced WESW’s decisions to transition from sex work

Irrespective of the specific decision made (considering leaving sex work, leaving sex work, or continuing with sex work), the decisions of WESW to transition were influenced by a variety of individual-level factors. We operationalized “Individual level factors” as those factors that pertain to the internalized experiences and characteristics of WESW themselves. WESW’s narratives focused on factors which included anticipated stigma, discrimination and age.

### Anticipated Stigma and Discrimination

Anticipated stigma and discrimination refer to the expectation or anticipation of experiencing stigma or discrimination based on a stigmatized characteristic or status (Lazarus et al., 2012). In the context of sex work, if a sex worker feels that society might judge them or hold prejudiced views because of the nature of their work, they might experience anticipated stigma and discrimination. This anticipation can lead to internalized negative feelings, such as shame, anxiety, or a sense of being devalued, even if direct discrimination or stigma has not occurred. With some of the WESW who felt anticipated stigma and discrimination, they expressed feelings of embarrassment, societal condemnation, and disrespect associated with their work. For example, Kateri who was considering leaving sex work, mentioned that; *I am deciding to do another job because this job is embarrassing*. For Fatumah who was also considering leaving sex work, she felt that sex work is perceived as an evil in her community; *Currently I am considering transitioning to another job if I happen to get it because working as a Neko (to mean sex worker) is perceived as evil*. Other WESW felt ashamed of themselves when people disrespected them or laughed at them as mentioned by Kirabo who too was considering leaving sex work; *They laugh at you; they disrespect you and you start feeling ashamed of what you are doing*.

Similar sentiments were made by Jessica, who mentioned that apart from being perceived as disrespectful, one would not even feel comfortable disclosing to their friends the type of work they were doing. She had this to say; *What can make me decide to quit the Neko job (to mean sex worker) is that it is not respectable, and you cannot tell your friends that “this is my job; “I sell sex” you see?*

Even the women who had decided to continue with sex work testified that, sex work was not a job they felt proud to do although they continued doing it. Rebecca who had decided to continue with sex work mentioned that she felt stigma engaging in sex work;

#### Rebecca

First of all, this isn’t a job that you can be proud of with other people apart from the fellow Nekos (to mean sex workers), if you happen to get another job that can be presented to others, you can stop being a Neko (to mean sex worker) and run the other job.

Helen felt that being a sex worker portrayed a bad image to the family or to community members if they got to know the type of work sex workers do. According to her, as much as she had decided to continue with sex work at the moment, she highlighted that it was a temporary job as she was looking for another job.

#### Helen

First of all, we have a bad image [from] the people who are not engaged in this work. My family members may get to know the type of work I do. Or one of the community members may come to get one of us on the streets and he sees me and goes back to gossip about me. That is why most of us wish to engage in this work for a short period of time as we look for other jobs.

### Womens’ age

Among those considering leaving sex work, age was frequently mentioned, while it was least mentioned among those who had already made the decision to leave sex work. None of the WESW who had decided to continue with sex work considered age as a determining factor in their decision-making process. When contemplating leaving sex work, participants emphasized that advancing in age would contribute to their decision, citing concerns about personal dignity and the type of work they would engage in as they grew older. They expressed that as they aged, so did their children, potentially causing shame. Additionally, participants noted that aging could impact their marketability, potentially leading to reduced payments from customers. Diana who was considering leaving sex work mentioned:

#### Diana

Things like age as each year that passes, I grow older which comes about with personal dignity. When children grow up and realize that [you are selling sex], it brings about shame. So, age might bring about quitting the job. And another thing, customers might reduce what they pay.

Angela was also concerned about how age, specifically old age, affected the market of WESW emphasizing that when one ages their market also reduced. This therefore influenced her decision to consider leaving sex work; “*Even the Nekos (to mean sex workers) have their market, as you age, the market reduces, so, that can also influence me to transition to other jobs”*. Mariam pointed out that as a WESW, she contemplated about her future and knew that one day she will grow old and must leave sex work; *“As you are silently seated, you think of the fact that you are growing up, and then make a decision to leave some jobs and transition to others.”*

For Sarah who had decided to leave sex work, she mentioned that she knew she was getting older and so did her children. She stated that her children may find out the type of work she was doing, and she decided to quit before that time came; “*Each and every day, I am getting older and also the children I am taking care of are also growing up.”*

### 2. Structural level factors

We operationalized “structural level factors” to encompass broader societal, economic, and environmental influences that shape the context in which sex work occurs. WESW’s narratives focused on factors which include interpersonal stigma and discrimination (from immediate family and community members), physical and sexual violence and income related factors.

### Interpersonal Stigma and Discrimination

Alongside the anticipated stigma and discrimination, some participants detailed experiencing interpersonal stigma and discrimination originating from their immediate family (parents/guardians), including their own children, and community members. Specifically, some women acknowledged that their involvement in sex work creates a negative perception of their character in the eyes of their children as they grow older. Other women expressed concerns that if their children became aware of their involvement in sex work, it might influence them [their children] to pursue or to engage in sex work as well. This was expressed by Anitah who was considering leaving sex work. Anitah shared that she did not want her children to be influenced by her and engage in the same type of work.

**Anitah**: *What can make me transition from being a Neko (to mean sex worker) is that whenever children see you doing that job, you feel ashamed since we go to work and come back in the morning. When children see you going at 8:00pm and coming back in the morning yet they have also grown up, they may be influenced to do the same. That’s what may influence my decision to get another job*.

Besides their own children, women stressed how their own parents and siblings could also add to the stigma and discrimination. They mentioned feeling uncomfortable about their parents or siblings knowing about their job because they were scared of being judged. For example, Gertrude, who made the decision to leave sex work, exemplifies how interpersonal stigma from parents and siblings played a crucial role in her choice. Gertrude explained that to avoid stigma from family members, some women engaged in other work, such as running a hair salon, to maintain a more respectable image. She mentioned:

#### Gertrude

Even our own parents feel bad whenever they hear about the kind of work, we engage in. But if let’s say a sister comes to visit you and they meet you at a saloon, it portrays a better picture than meeting you in a small room which brings so many questions about the kind of work you do…

In addition to the stigma from family members, women also faced stigma from the community where they lived. Women mentioned experiencing disrespect and being excluded from community gatherings or opportunities to contribute. Some women mentioned that when people found out they were involved in sex work, even distant acquaintances reacted with disgust, yet some individuals who showed contempt were actually from a lower social status than the women themselves. For example, Cecilia, who was considering leaving sex work, shared a poignant insight, saying, *“You might get despised by a person who shouldn’t have despised you, but because you are a Neko (to mean sex worker)*……”

Other women shared experiences where they felt dehumanized due to their involvement in sex work. They expressed how the communities they lived in associated sex work with immense stigma and discrimination. These women faced constant judgment and were viewed as belonging to a lower social class, lacking the respect they deserved despite contributing significantly to society. Grace who was considering leaving sex shed light on this saying; *“This job is embarrassing and makes you disrespected in the community and that is why most of us feel shy and act as if we are mentally ill. You feel frustrated*.”Matilda, another woman considering leaving sex work echoed similar sentiments regarding dehumanizing treatment in the community, conveying that they are not seen or respected as fellow human beings; *“People talking about you and treating you like you are not human*, *that you are just Neko (to mean sex worker). That’s why I work hard so that I can quit that job”*.

Other women who had decided to leave sex work expressed feeling marginalized within their communities, citing instances where their legitimate issues, such as falling ill, were disregarded, and they were denied fair consideration and treatment. This dehumanizing treatment played a pivotal role in their decision to exit sex work. Regina provided the following insight;

#### Regina

A Neko (to mean sex worker) does not bring up suggestions, cannot sit in any meeting, people be like “what is that Neko suggesting” even when you get a problem, you have been attacked by a sickness and fallen, they will say that “Neko was drunk” even [though it was never the case]…”

### Physical and Sexual Violence

WESW experience various forms of physical violence from their clients, ranging from instances of strangulation to attempted murder. Additionally, some WESW experience sexual violence in the form of attempted rape perpetrated by clients. These risks significantly contribute to negative health consequences for WESW, such as exposure to STIs and HIV, unintended pregnancies, abortion, as well as emotional and mental health issues. These risks also serve as major factors compelling WESW to turn to alcohol and other drugs as coping mechanisms while engaged in sex work.

Regardless of the decision taken, WESW consistently reported instances of physical and sexual violence, which served as both significant barriers and motivating factors for their transition from sex work. For example, WESW who considered leaving sex work mentioned incidences of rape and torture from clients like being strangulated. Similar issues were raised by WESW who had made the decision to leave sex work. Some of these women had also experienced theft by customers, non-payment by customers, and physical fights initiated by customers. Even among WESW who had chosen to continue with sex work, similar experiences were acknowledged and recounted.

Anitah who was considering leaving sex work reported an incident where a client attempted to rape her. Such an incident left emotional and physical pain as she narrates; “A customer has ever attempted to rape me and, in this attempt, I was teared. So, when I think about that situation, I wish I could have another job to do other than that [sex work].”

Multiple cases of client-inflicted torture were reported by the women, involving distressing acts such as strangulation. Four women who were considering leaving sex work shared similar experiences. Theresa disclosed instances of torture and non-payment by clients, despite engaging in sexual activities with them. Theresa also highlighted incidents where clients refused to use condoms; *“Neko business (to mean sex work) involves torture*, *sometimes*, *a customer refuses to pay even after having sex with you, yet he has affected your life. Other times he refuses to use condoms even when you ask him to use them”*

Ruth reported about experiences of being strangled inflicted by clients; “One major factor that could make me leave sex work is that it has a lot of problems, many women lose their lives to being strangled and killed. Beatrice and Lucy shared similar sentiments respectively; The problem in it is getting a customer after agreeing, when you get there…[he] wants to use you without giving you any coin, sometimes in such a bad situation and after using you, then wants to kill you (Beatrice). Lucy recounted a similar experience; Being a Neko (to mean sex worker) we encounter many challenges like you can be killed. This can be done by the customers or else can be sent by another person to kill you.

For women who decided to leave sex work, they were primarily motivated by the physical and sexual violence perpetrated by their clients. Women who chose to leave sex work shared comparable experiences to those who were considering leaving sex work. These experiences included instances of theft by customers, non-payment for services rendered, attempted rape, and physical fights initiated by customers. The cumulative impact of these risks played a significant role in their decision to transition from sex work.

Regina reported about how some customers refuse to use condoms in addition to stealing from them. She lamented; “The way we are treated; you can bring in a man but does not want to use a condom you take him into a room, he has caused harm to you, has stolen your money There are many things associated with prostitution”.

Nadia, who made the decision to leave sex work, shared her personal experience, which likely played a role in shaping her choice to leave sex work; *“There are many reasons I would leave that job; you may be coming from there*, *and they rape you.”*

For Ritah who also had decided to leave sex work, she narrates similar experiences of physical violence from clients who at the same time didn’t want to pay:

#### Ritah

We were always facing challenges previously. You could get a man when he is drunk and he would want to take you [have sex with you] for free, sometimes he would use you and not pay you or he fights you after using you. So, I decided to find ways of leaving the job.

### Income related factors

Among the structural level factors that influenced the decisions of WESW to transition from sex work, income emerged as another predominant factor. The need for financial resources was articulated in diverse ways, for example the need for start-up capital, gaining financial stability, lower returns on income, getting an alternative source of income, and planning for future prospects.

Women who were considering leaving sex work expressed the need to remain engaged in sex work to accumulate start-up capital and achieve financial stability. These women mentioned that they had plans to start small businesses to cater for basic needs like rent, take care of their children, and sustain their own lives. For example, Mariam, Faridah, and Theresa were looking for start-up capital to start small businesses which could support them and take care of their children; *“If I can start up my own stall, and it can provide me with enough income to pay for rent*; *take care of my children and myself, then I can leave sex work (Mariam)”*. Faridah had similar plans and mentioned; *“For when I get money, I can start a business so that I can sustain myself”*. For Theresa, she wanted to accumulate enough capital so she could be self-employed; *“Whatpushes me to work as a Neko (to mean sex worker) is that once I accumulate enough capital for me to be self-employed, I will quit sex work”*.

Some women reported engaging in sex work not out of choice, but rather due to financial constraints. For example, Afiya mentioned that she didn’t engage in sex work because she wanted to but simply because of the situation she found herself in:

#### Afiya

Personally, I engage in this work because of the situation I find myself in. But if I get capital, I will start up a business where I can earn income so that I am able to take care of my children and leave sex work. For sure this is not the kind of job I am proud of, or what I can share with my friends.

Like WESW who were considering leaving sex work, WESW who had decided to continue with sex work, had similar financial constraints that worked as major barriers to leave sex work. These women expressed the need for start-up capital, getting an alternative source of income or job, while others mentioned that they were still planning for the future. Women who were still looking for start-up capital had plans to start and expand their business which could supplement the income they get from sex work. For example, Betty and Josephine mentioned that, if they got start-up capital, then they would leave sex work. *“Getting capital, which capital is enough for me or when my business expands, there I can I quit sex work and concentrate on my business (Betty)”* Josephine had similar sentiments; *“I can leave sex work if I get enough capital, start up my business and get enough money to meet my needs”*.

Other women who had decided to continue with sex work mentioned that they could leave sex work if they got an alternative source of income or a job. For instance, Lydia and Winnie, although they had decided to continue with sex work mentioned that if they got another job where they could earn from, then they could leave sex work. “*If I get another job that I earn income from then I drop the sex work job (Lydia)”*. Winnie had similar plans; *“What may cause me to transition is when I get a job”*.

Amina who had decided to continue with sex work highlighted that, she was still planning for the future. Her statement also indicates that although her current decision was to continue with sex work, it was only conditional to planning for future prospects; *“I am preparing myself and I am completing building my house such that by the time I quit, I am not a tenant, and also when my children are educated (Amina)”*.

While women who were considering leaving sex work and those who had decided to continue with sex work shared similar financial constraints, Nadia who decided to leave sex work felt that she had not gained much from sex work. She felt that with lower returns on income, she had decided to leave sex work; *“I have not gained much from my time as a Neko (to mean sex worker); when you get money, it vanishes when you have also contracted diseases (Nadia)”*.

In conclusion, the decision-making process of WESW regarding transitioning out of sex work is multifaceted, shaped by a complex interplay of individual and structural factors. At the individual level, experiences of anticipated stigma and discrimination, coupled with the challenges of aging, emerged as prominent catalysts prompting many WESW to contemplate or actualize their transition from the sex work industry. The burden of stigma not only affects the self-esteem and mental well-being of WESW but also extends to their familial relationships, with concerns about the well-being and future prospects of their children playing a significant role in their decisions.

Moreover, at the structural level, pervasive issues such as physical or sexual violence, interpersonal stigma and discrimination, and economic instability exert profound influences on WESW’s trajectories out of sex work. These structural factors create a hostile environment that not only perpetuates the vulnerabilities of WESW but also restricts their agency in seeking alternative livelihoods. The confluence of these challenges underscores the urgent need for comprehensive interventions that address both individual and structural barriers faced by WESW.

## Discussion

This study explored the decision-making process related to transitioning from sex work into other jobs among WESW in Uganda. We identified three primary types of decisions that WESW took in their transitioning journey from sex work, that is WESW who were still considering leaving sex work, those who had decided to leave sex work, and those who decided to continue doing sex work. A number of individual and structural level factors influenced the WESW’s decisions to transition from sex work. At the individual level, results indicated that the majority of WESW were either considering leaving sex work or had decided to leave sex work. The major drivers of these decisions were anticipated stigma and discrimination and age while at the structural level, issues of physical and sexual violence, interpersonal stigma and discrimination, and income related factors emerged as the major barriers to transition from sex work.

The concept of stigma and discrimination as explored in this study at both the individual and structural levels, underscores a pervasive psychological burden faced by WESW. The expectation of prejudice and judgement, irrespective of direct or indirect experiences of stigma and discrimination, can catalyze a cycle of self-stigmatization. This internalization of societal values manifests as profound emotional distress, potentially impacting mental health well-being of WESW. Consistent with previous studies, WESW continue to face different forms of stigma and discrimination and are commonly seen as deviant from the social norms of society (Benoit et al., 2018). WESW face stigma and discrimination from family members and the communities they live in, (Dunkle et al., 2005; Scorgie et al., 2012; Udoh et al., 2009). This greatly impacts their mental health (UNAIDS, 2021b), family relationships (Davey et al., 2018; Kiyingi et al., 2023; Lazarus et al., 2012), participation in communities, and access and utilization of health services such as HIV testing and treatment (Scorgie et al., 2013). Lack of utilization and access to health services in turn exacerbates WESW’s burden of HIV, risk of HIV transmission and poor treatment outcomes (Camlin et al., 2020; Davey et al., 2018).

The role of age in the decision-making processes of WESW was another critical factor with profound implications for their personal and professional lives. Our study revealed that advancing in age (for sex workers) is not merely a chronological marker but an important point that intersects with social norms, economic realities, and personal dignity, influencing the decision to transition out of sex work. According to the experiences shared by WESW, advancing in age affected how these women perceive their future within the industry and their potential for employment outside of it. WESW’s experiences highlighted that older sex workers may experience a decline in demand for their services due to societal ageist attitudes, which can impact their earning potential and economic stability. This economic pressure, coupled with the desire for personal dignity and societal respect, can influence WESW’s decision to transition out of sex work. Age can also intersect with social norms that stigmatize and marginalize WESW, especially as they get older making it challenging for older sex workers to integrate into other professions or social networks.

Our study findings also highlighted the intersection of physical and sexual violence with sex work which also raises profound questions about the safety and well-being of WESW. This study has illuminated the disturbing reality that such violence is not an anomaly but a pervasive risk that WESW encounter regularly. The implications of these experiences are multifaceted, affecting physical and mental health and well-being of WESW (Oram et al., 2012), and influencing the critical decisions of whether to continue with or leave sex work. Similar to our study findings, instances of physical and sexual violence, encompassing physical fights and rape, have been previously documented by WESW in a study conducted by Witte and colleagues in Mongolia (Witte et al., 2010). Physical and sexual violence experienced by WESW contribute to a heightened risk of STIs and HIV, unintended pregnancies, and the necessity for abortions, which are significant health burdens (Phrasisombath, Thomsen, et al., 2012; Witte et al., 2010,^2011^). Benoit and colleagues highlighted that given the high frequency of sexual risk-taking behaviors among WESW (Benoit et al., 2018), they have the highest HIV prevalence rates when compared to the general population globally and in Uganda as well (UNAIDS, 2021b, 2021a). Moreover, the emotional trauma accompanying such violence can lead to long-term psychological distress, including post-traumatic stress disorder (PTSD), depression, and anxiety.

The recurrence of violence in the narratives of WESW, regardless of their future intentions within the industry, suggests that violence is a systemic issue rather than isolated incidents. It indicates a need for structural interventions, including legal reforms to protect the rights and safety of sex workers, and the establishment of support systems for victims of such violence. The coping mechanisms that WESW adopt to, often involving alcohol and other drugs (Li et al., 2010; Roshanfekr et al., 2015; Witte et al., 2010) point to the inadequate support and resources available to them. These strategies, while providing temporary relief, may further compound their health risks and socioeconomic vulnerabilities (Phrasisombath, Faxelid, et al., 2012; Sanders, 2004; Spice, 2007; Wechsberg et al., 2006).

In addition, the decision to leave sex work, as influenced by experiences of violence, reflects a crucial survival strategy. It is a response not only to immediate harm but also to the anticipation of future risks. This anticipation of harm plays a critical role in decision-making processes and highlights the need for alternative employment opportunities that offer safety and dignity to WESW. The study findings call for a comprehensive approach to addressing violence against sex workers. This includes policy changes that decriminalize sex work and destigmatize those within the industry, community-led initiatives to provide support and advocacy, and healthcare services that are accessible and sensitive to the needs of WESW. Moreover, experiences shared by WESW in this study suggest that societal attitudes toward sex work contribute to a culture of impunity where violence against WESW is normalized as highlighted by Caldwell and de Wit (2021). This normalization of violence has deep societal implications, as it not only affects the individuals involved but also reflects and reinforces gendered power imbalances and societal perceptions of WESW.

In terms of income-related factors, this was a common factor influencing women’s decisions to transition from sex work. This factor was commonly mentioned by women who were considering leaving sex work and those who had decided to continue with sex work. The relationship between economic necessity and engagement in sex work is complex and multifaceted, reflecting the broader socioeconomic conditions that often limit the choices available to WESW. The necessity for financial resources, articulated through the need for start-up capital and financial stability, highlights a significant barrier to exiting sex work. WESW’s aspirations for financial independence through entrepreneurship or stable employment are often hindered by economic constraints faced by women in general. Research has emphasized that financial stability, achieved through a supportive relationship, access to bank loans, financial aid for starting a business, or assistance from friends or relatives, is a critical factor in facilitating the transition out of sex work (Mazeingia & Negesse, 2020; Menezes, 2019). This underscores the critical role of economic empowerment interventions (Ssewamala et al., 2019) and the availability of capital as determinants in the potential transition away from sex work.

The drive for financial stability is not only motivated by the individual WESW’s needs but also by the responsibilities they hold, such as providing for children and securing housing or shelter. This dual pressure of personal and familial financial obligations indicates that economic incentives are deeply intertwined with social and familial roles, reinforcing the centrality of income in WESW’s decision-making process. However, the planning for future prospects, such as starting a business or expanding existing ventures, illustrates a forward-looking strategy among WESW. This forward planning is, however, contingent upon access to financial resources, which can be a significant barrier for many WESW. The lack of access to capital and financial services for WESW is a reflection of broader structural inequities that must be addressed to facilitate successful transitions out of sex work.

Recognizing the dynamic interplay between individual agency and structural constraints is crucial for designing effective policies and interventions aimed at supporting WESW in their transition out of sex work. Efforts to mitigate the impact of stigma and discrimination should be accompanied by initiatives addressing the root causes of violence and economic marginalization within the sex work industry and women in general.

Thus, supporting women transitioning from sex work, interventions and policies must adopt a multifaceted approach. This includes advocating for legal reforms to decriminalize sex work and destigmatize those involved, as well as enabling protection from all forms of violence (Jewkes et al., 2023). Decriminalization of sex work is foundational for enabling safer working conditions and better health outcomes for WESW (McCann et al., 2021). In addition, there should be accessible healthcare services sensitive to sex workers^'^ needs, along with safety measures and support systems, are crucial to address physical and mental health consequences of violence and stigma experienced by WESW (Jewkes et al., 2023). Further still, addressing societal attitudes through awareness campaigns and integrating sex workers into broader social networks fosters a supportive environment for transitioning into other professions.

Furthermore our findings emphasize the need for economic empowerment interventions, in terms of savings-led microfinance, vocational and skills training, and entrepreneurship programs, tailored specifically for WESW (Ssewamala, Han, et al., 2010; Ssewamala, Ismayilova, et al., 2010). Such interventions would provide the necessary resources to overcome the financial barriers to exiting sex work, by empowering WESW to make sustainable transitions into alternative professions. Economic empowerment interventions specifically those tailored to savings-led microfinance can reduce the stigma and discrimination faced by WESW by creating new identities as entrepreneurs or skilled workers (Witte et al., 2010,^2011^,^2015^) thereby integrating them into different societal roles that are more socially accepted. Furthermore, with financial resources, WESW are better positioned to leave environments where they are susceptible to violence and seek safer, more secure living conditions for themselves and their children. This shift can enhance their personal agency, contribute to a sense of dignity, and allow for the pursuit of personal development and growth.

In the context of the Uganda Government Vision of 2040 which focuses on the financial inclusion of vulnerable groups, including WESW (Ssewamala et al., 2019), economic empowerment interventions are aligned with broader development goals. Ensuring that WESW are included in financial growth strategies not only helps them individually but also contributes to the economic and social development of the nation as a whole. These structural interventions thus act as a bridge, providing the necessary support and resources at the macro level to address and mitigate the challenges faced at the micro or individual level.

It is important to acknowledge that other factors such as stigma, discrimination, aging, and experiences of violence are deeply interwoven with economic stability and opportunity. Without financial security, the ability of WESW to leave sex work is significantly constrained, as the risk of poverty and inability to provide for themselves and their families can perpetuate their involvement in sex work despite the presence of these barriers and motivators. This comprehensive approach can enable WESW to make a sustainable transition out of sex work and into alternative professions where they can thrive.

### Strengths and Limitations

Our study highlights significant findings about the complexity and multifaceted decision-making among WESW to transition from sex work. The study’s emphasis on individual and structural factors provides a comprehensive understanding of the complexities involved in the transition from sex work to other vocations. By focusing on stigma and discrimination, age, physical and sexual violence, and income as key influences in the decision-making process, the research offers valuable insights into the psychosocial and economic dynamics that drive WESW’s decisions. The consideration of stigma and discrimination, particularly, amplifies the psychological burden and societal challenges faced by WESW, highlighting an area that is often overlooked in research. The inclusion of age as a factor sheds light on the intersectionality of sex work with societal norms and economic realities, offering a nuanced perspective on the life course of WESW. The study’s findings on violence provide critical data on the risks associated with sex work, reinforcing the need for structural interventions and support systems that target WESW. The emphasis on economic factors, particularly the necessity for financial resources and the barriers to financial independence, aligns with broader socio-economic challenges that women face in general in developing countries.

Despite its strengths, the study also has some limitations. One major limitation of this study is that the analysis and data collection were not originally targeted explicitly on the issue of women’s transition from sex work. Thus, future research should be done with more comprehensive questions on women’s decisions to transition from sex work. This study analyzed data from one time point. Longitudinal data should be analyzed to examine WESW’s trajectories with transitioning from sex work. Although the research provides a thorough analysis of the various influences on WESW’s decisions, it may not fully capture the diversity of experiences among sex workers in different regions or with different socio-economic backgrounds. Moreover, the study is geographically limited to Uganda, which may limit the generalizability of the findings to other contexts, particularly where legal frameworks and societal attitudes towards sex work differ significantly.

## Conclusion

Our study’s findings highlight how challenges like stigma, discrimination, aging, and violence are intricately linked with the struggle for economic autonomy for WESW. These adversities not only precipitate the doubt that WESW have to exit sex work but also underscore the urgency for WESW to seek alternative livelihoods that offer both financial stability and freedom from the current occupational identity.

The quest for alternatives, as posited by the role exit theory and observed in our findings, is not a sole endeavor for WESW but one that necessitates robust support systems and resources. The exit from sex work, laden with potential psychological, financial, and social consequences, is navigated with varying levels of support, reflecting the theory’s recognition of the differential access to resources during role transitions. Our conclusions advocate for multifaceted interventions and policies that address the different challenges that WESW face as they make the decision to transition from sex work.

## Figures and Tables

**Table 1:** Participant demographic characteristics-Kyaterekera qualitative interviews-Time 2 (N = 53)

Variable	Total Sample (N = 53)
	(n) %
**Age** (Mean, SD) (min/max = 20–47)	31.79 (6.42)
**Marital status**	
Married	5 (9.43)
Divorced	31 (58.49)
Separated	4 (7.55)
Widowed	2 (3.77)
In a relationship	8 (15.09)
Single, never married	3 (5.66)
**Religion**	
Catholic	27 (50.94)
Protestant	17 (32.08)
Muslim	9 (16.98)
**Highest level of education**	
Did not go to school	6 (11.32)
Dropped out before primary 7	20 (37.74)
Completed primary 7 and stopped	10 (18.87)
Dropped out before senior 4	3 (5.66)
Technical/vocational college	1 (1.89)
**Household size**	
Number of people in the household (mean, SD) (min/max = 1–8)	3.07 (1.68)
Number of children in a household (mean, SD) (min/max = 0–6)	1.66 (1.52)
**Monthly income**- UGX (mean, SD) (min/max = 10,000–700,000)	205,037.7 (171,041.6)
**HIV Results**	
Positive	19 (35.85)
Negative	34 (64.15)
STIs	4 (7.55)
Duration of sex work in years (mean, SD) (min/max = 0.66–23)	6.44 (5.01)
Mean age in years at start of sex work (mean, SD) (min/max = 14–42)	25.49 (6.26)
Number of different customers in the last 30 days (mean, SD) (min/max = 2–238)	28.41 (42.30)

## Data Availability

Data used in this analysis is available upon reasonable request, data access requests can be sent to any of the following Associate Deans-at Washington University’s Brown School. Provided the conditions outlined below are met, there should not be concern about data sharing. The team is open to data sharing provided the points outlined below, which were part of the study protocol, data sharing plan, and consenting process, are met. Siomari Collazo-Colón, JD, Associate Dean for Administration, Hillman Hall, Room 254, [o] 314.935.8675 [f] 314.935.8511, Brown School | Washington University in St. Louis, [e] scollazo@wustl.edu OR Fred M. Ssewamala, PhD [Study Co-PI], William E. Gordon Distinguished Professor, Associate Dean for Transdisciplinary Faculty Research, Professor of Medicine, Washington University School of Medicine, Goldfarb, Room 343, Brown School Washington University in St. Louis, [o] 314.935.8521 [e] fms1@wustl.edu. A formal research question is specified a priori. Names, affiliations, and roles of any other individuals who will access the shared data; The deliverable(s)—e.g., manuscript, conference presentation-are specified a priori; Proper credit and attribution—e.g., authorship, co-authorship, and order—for each deliverable are specified a priori. A statement indicating an understanding that the data cannot be further shared with any additional individual(s) or parties without the Pi’s permission; IRB approval for use of the data (or documentation that IRB has determined the research is exempt). The requestors are expected to handle converting electronic formats (though the research team will consider converting to tab-delimited text format if possible). These conditions were prespecified in our study proposal, study protocol data sharing plan, and consenting and assenting process. Participants enrolled in the study are vulnerable women engaged in sex work, and over 40% of them living with HIV-both highly stigmatized. Thus,
